# Listening with Humility:Lessons Learned from a Dialogue with Scholars from Critical Autism Studies

**DOI:** 10.1007/s40617-025-01149-7

**Published:** 2026-01-09

**Authors:** Sneha Kohli Mathur, Jonathan Tarbox, Nick Chown, Elsa Suckle

**Affiliations:** 1 University of Southern California, Los Angeles, CA USA; 2 University of Southern California and FirstSteps for Kids, Los Angeles, CA USA; 3 Independent Scholar, Birmingham, UK; 4 Bath Spa University, Bath, UK

**Keywords:** Applied behavior analysis, Critical autism studies, Critical behavioral studies

## Abstract

The field of applied behavior analysis (ABA) appears to be at an inflection point where we are experiencing substantial criticism from the autistic community. We as a field can choose to defend our field from the criticism or we can choose to listen and be responsive to it. Some early forays into discussing the implications of neurodiversity for ABA have been fruitful (Veneziano & Shea, 2023; Mathur et al., 2024) and it seems clear that the time for direct dialogue between ABA scientist practitioners and some of our greatest critics has come. Suckle et al. (2025) described one side of a recent dialogue between scholars of ABA and Critical Autism Studies (CAS), in which CAS scholars posed questions to ABA scholars and ABA scholars answered them. That article was explicitly composed for a disability studies audience and accordingly published in a disability studies journal. The current article describes the other side of that dialogue, in which ABA scholars posed questions to CAS scholars, who then provided their answers. The current article is explicitly written for the ABA researcher and practitioner audience. We may not feel entirely comfortable with some of the criticisms of ABA that come from CAS scholars but we believe that willingness to experience this discomfort is a critical prerequisite for our field to evolve. This article explores how our field can engage in cross-disciplinary collaboration and concludes with potential actionable steps that ABA researchers and practitioners can put into practice today.

Prominent scientist practitioners in the field of applied behavior analysis (ABA) have long called for the field to engage in self-reflection and evolution, with the overarching goals of our field becoming more effective and more ethical on an ongoing basis. For example, nearly 50 years ago, Holland (1978) called for researchers to consider whether behavior modification procedures were being used in ways that actually uplifted vulnerable communities or whether they were being used to gain compliance from those who lacked the power to self-advocate. In 1989, Sidman called for the field of behavior analysis to turn away from coercive procedures, such as the punishment procedures that were commonly used and endorsed by some of the field’s most visible researchers (e.g., Lovaas, 1987). As mistreatment of developmentally disabled clients by professionals from a variety of fields, including behavior analysts, became public knowledge in the 1980s and 1990s, calls were made for the field of ABA to organize into something resembling a professional discipline and to focus on establishing ethical guidelines (Bailey & Burch, 2016). In more recent decades, scholars have called on behavior analysts to work toward greater equity for women (Ruiz, 1998; Li et al., 2019) and racial justice (Gingles et al., 2022) in the field of ABA. The *Ethics Code for Behavior Analysts* recently called for behavior analysts to attend to client assent (Behavior Analyst Certification Board [BACB], 2020) and authors have begun to outline ways in which client assent can be honored during service delivery, even when clients do not possess the speaking repertoires to give their assent vocally (Breaux, 2023). In short, for decades, leaders in the field have called for behavior analysts to be proud of our strengths, but also to humbly acknowledge our shortcomings and do better (Neuringer, 1991).

Researchers and practitioners in ABA have begun responding to criticisms of ABA coming from the neurodiversity movement. In what was perhaps the first discussion of neurodiversity in a peer reviewed article in a behavior analytic journal, Veneziano and Shea (2023) discussed the concern from the autistic community that ABA programs appear to be attempting to make autistic clients appear “normal.” Although many ABA practitioners (we use the term “many” in this article not to refer to any quantified or empirically established proportion) may reject this notion, Veneziano and Shea connected this concern to the seminal Lovaas (1987) article, which explicitly advocated for the goal of making clients indistinguishable from their peers. The authors discussed how that goal was likely socially invalid from the standpoint of the autistic clients themselves, and how this early mindset likely continues to have an influence on how goals are selected in ABA supports for autistic clients to this day. Expanding on the need to abandon indistinguishability as a goal of ABA services for autistic people, Graber and Graber (2025) suggested taking an additive approach, as opposed to a subtractive approach. In particular, they advocated for ABA services to conceptualize the goal of supporting autistic clients to consist of teaching new skills that expand learner’s repertoires, rather than focusing on eliminating behaviors that may appear autistic.

Allen et al. (2024) expanded the conversation on neurodiversity and ABA by discussing how ABA research and services for autistic people might be reevaluated by considering the contributions of the fields of disability studies, including the perspective of disability as a form of diversity. The authors discussed how ABA might evolve to become more neurodiversity-affirming by more carefully considering client identity, refocusing on client dignity and self-determination, as well as reevaluating social validity in ABA services, from the standpoint of the autistic clients receiving those services.

Behavior analysts have begun empirically investigating the social validity of ABA procedures from the perspective of autistic people, as the original conception of social validity in ABA requires (Wolf, 1978). Chazin et al. (2024) conducted a survey of 226 autistic adults, wherein respondents rated their acceptability of a variety of common ABA goals and procedures. The study assessed a large variety of goals that are commonly addressed in ABA programs for autistic people, with some of the goals potentially being more relevant to learners who are autistic with co-occurring intellectual disability, as opposed to “only” being autistic. Therefore, it may be somewhat difficult to disambiguate the autistic social validity of goals for autistic learners, versus goals for learners with intellectual disability. However, the overwhelming majority of the participating autistic adults said that goals of increasing eye contact and decreasing stereotypy—both of which can be harmful—should either “never” be taught or should be “very low priority.” Results showed that autistic adults approved highly of goals of increasing communication and self-advocacy skills, such as learning to say “stop” or “no.” In terms of procedures, respondents highly approved of communication devices and procedures that accommodated sensory needs and emotional safety, although disapproving of arbitrary reinforcers (e.g., edibles and tokens) and classroom-wide punishment systems.

Mathur et al. (2024) reviewed criticisms of ABA interventions from the neurodiversity movement, including: (1) erasing autistic identity; (2) overfocusing on compliance as a goal; (3) reductionistic focus on overt behavior and common behavioral functions; (4) autistic voices are absent in ABA research; and (5) practitioners insisting on ABA as the only treatment choice. In response to each potential criticism, the authors attempted to discuss examples of how the criticism can be used as points of self-reflection to evolve ABA research and practice with autistic clients.

Shortly after the Mathur et al. (2024) article was published, the first and second authors of the current article received an email from two respected scholars in the CAS field, Nick Chown and Elsa Suckle (third and fourth authors of the current article). The email exchange quickly turned into conversations over video conference, which allowed the team to exchange knowledge and strive to understand each other’s perspectives, including potential hurdles to communicating across the disparate fields. With mutual respect and the goal to build mutual understanding across our respective fields, we have turned these conversations into two collaborative articles. The first was composed for the CAS community, interrogating how ABA could strive to do better and engage with ABA practitioners to explore if and how ABA can be autism-affirming (Suckle et al., 2025). In that article, we attempted to show how scholars from CAS can engage ABA scholars with questions that probe areas of potentially grave concern, and how ABA scholars can strive to respond to those questions with humility. We believe that article demonstrated productive, peaceful dialogue that managed to produce some clarity around points of agreement between ABA and CAS.

Jackson-Perry (2025) cited the Suckle et al. (2025) article as demonstrating some elements of the research practice they coined as “unknowing.” Unknowing is described as a process of approaching a research topic with the assumption that one’s mainstream knowledge in that topic area is fallible. For example, as ABA scholars, practicing unknowing would include starting with the assumption that behavior analytic scientific knowledge of autism is necessarily incomplete, especially considering the extent to which that knowledge has been produced in a manner that has not included autistic researchers and scholars from other disciplines studying autism, including CAS. Of course, when engaging in interdisciplinary collaboration, unknowing involves a two-way street, in which researchers from other disciplines (e.g., CAS) would begin by adopting the assumption that the knowledge that they have of ABA likely is not complete, because the creation of that knowledge may not have included scholars of ABA. Furthermore, the practice of unknowing explicitly calls for researchers of all backgrounds to intentionally practice sitting with discomfort and curiosity through the research process, rather than seeking to confirm their existing knowledge (Jackson-Perry, 2025).

The current article is the second in the two-article series that was produced by the CAS–ABA dialogue between the four authors of this article. Although the first article was composed for the CAS audience, this article is explicitly composed for the ABA research and practice community, in hopes of promoting the benefits of feedback from CAS for making our field more autism-affirming. The larger goal in this program of collaboration is to discuss the importance of cross-disciplinary collaboration and how to do so in a manner that is mutually respectful and productive.

## Language

Although neurodiversity or “neurodiversity affirming care” have become terms commonly used, sometimes as marketing tactics, understanding the definition of neurodiversity is important. *Neurodiversity* in and of itself is simply a biological fact. Further, it is a noncontroversial fact that differences in brain structure and functioning among people affect how people experience the world (Singer, 2016; Botha et al., 2024). Beyond the biological fact that neurodiversity exists, there is disagreement on what neurodiversity encompasses, for example, whether or not intellectual disability is an aspect of neurodivergence. However, generally speaking, the term *neurotype* is used to refer to a group of people who share particular neurological structures and functions that affect the way in which they interact with their environment in meaningful ways. When used to refer to groups of people who have neurotypes that differ from the predominant group, the term “neurodiversity” often includes autism, attention deficit hyperactivity disorder, and communication disorders, among others. The term *neurotypical* refers to people whose neurological structure and function is within the “average” of the larger population (Rosqvist et al., 2020). The *neurodiversity paradigm* is an academic field that approaches differences in neurotype as a form of divergence, not deficit (Rosqvist et al., 2020). The term “neurodiversity affirming care” is generally used to refer to care for neurodivergent individuals that respects their neurotype, incorporates input from individuals in the community, and seeks to support, rather than cure, individuals. The possibility of whether ABA can be neurodiversity affirming remains a controversial topic; however, some behavior analytic researchers have begun to suggest steps for evolving ABA toward being more neurodiversity affirming, as discussed above (Allen et al., 2024; Chazin et al., 2024; Graber & Graber, 2025; Mathur et al., 2024; Veneziano & Shea, 2023).

## Conceptual and Historical Context

The dialogue that forms the heart of this article comprises an interaction between researchers from the fields of CAS and ABA. Brief context is provided for each discipline below.

### Critical Autism Studies

Critical Autism Studies is a relatively new transdisciplinary academic field that studies how societal and cultural influences shape the experiences of autistic people. CAS examines how and if the medical model of disability actually represents the lived experiences of autistic people, and strives to take a deeper look at the intersectionality (e.g., the various aspects of a person’s identity, such as race and gender) of an entire person with respect to treatment and support, rather than just the behaviors a person engages in (Milton & Ryan, 2023). Furthermore, CAS investigates “power dynamics that operate in discourses around autism, questioning deficit-based definitions of autism, and being willing to consider the ways in which biology and culture intersect to produce ‘disability’” (Waltz, 2014, p. 1337). Perhaps most important, the field of CAS is led by autistic people with the goal of creating socially just and inclusive communities that embrace neurodiversity (Milton & Ryan, 2023). Among the more important key positions of CAS is that professions who purport to serve autistic people must be educated about the lived experiences of autistic people and center autistic voices in service delivery. The clear implication of this foundational position is that any professionals serving autistic clients must be thoroughly trained on autism, especially knowledge that is created by autistic people.

## Applied Behavior Analysis

Broadly speaking, behavior analysis started with a humanistic vision of creating a world free from coercion (Skinner, 1948). Yet as behavioral principles began to be implemented with the human population, especially with disabled individuals and individuals with severe mental illness, the use of aversive behavior modification procedures became common (Bailey & Burch, 2016). The early application of behavior modification to autistic children was replete with harsh aversives and perhaps the most influential behavioral publication on autism treatment explicitly called for the use of physical aversives (Lovaas, 1987). However, the more humanistic thread within behavior analysis never died and a variety of influential behavior analytic scholars called for treating people with dignity and respect throughout the early decades of the evolution of ABA research and practice. This includes the concepts of the constructional approach to ABA (Goldiamond, 1974), social validity (Wolf, 1978), social justice (Holland, 1978), Murray Sidman’s work against coercion (Sidman, 1989), and the positive behavioral supports movement (Horner et al., 1990). Decades later, we see contemporary echoes of these earlier calls for respecting human dignity in ABA in the areas of compassion (Taylor et al., 2019), trauma-informed care (Rajaraman et al., 2022), social justice (Gingles et al., 2022), and neurodiversity-affirming ABA (Graber & Graber, 2023; Veneziano & Shea, 2023), among others. The authors of this article consider this dialogue to be one that is based on the humanistic values that formed the historical foundation of ABA, while making contact with and acknowledging harmful practices of the past and present. The current dialogue, then, might be considered part of the contemporary movements within ABA that center on human dignity. Furthermore, the *Ethics Code for Behavior Analysts* (BACB, 2020) explicitly calls for behavior analysts to engage in cross-disciplinary collaboration and so this dialogue between the disciplines of CAS and ABA may be considered an attempt at forging the beginnings of such collaboration between two fields that have historically been at odds with one another.

## Designing a Structured Dialogue

### Contact Hypothesis

The contact hypothesis (Allport, 1954) describes peaceful and respectful interpersonal interaction as an approach to building understanding and thereby reducing stereotyping, discrimination, and prejudice between majority and minority groups. The contact hypothesis encourages interpersonal contact and dialogue to increase collaboration between people from different backgrounds, in this case bringing autistic and non-autistic people with an interest in ABA-based interventions to the table.

The motivation for the behavior analyst authors on this article was to increase collaboration between ABA professionals/scholars and some of our field’s strongest critics. Many in the ABA field have expressed concern over the ubiquitous public criticism of ABA and the potential negative consequences that it may have for our profession, as well as our clients’ access to ABA services. However, this team was motivated to increase respectful collaboration between the ABA and autistic communities for another reason that we believe is even more important: Amplifying and collaborating with the voices of those we serve is morally and ethically imperative, regardless of what practical outcomes it may or may not produce. In addition, our forays into dialogue with autistic adults in recent years revealed a picture that was very different from the overly simplified stereotypes that some ABA professionals contact on social media. Rather than critics who are fixed in their judgments about ABA, what we observed were thoughtful, reasonable people who cared about the same things we did, supporting autistic people to thrive and live their best lives. Based on these small initial successes, we believed that building respectful collaboration and knowledge-sharing with autistic advocates who were critical of ABA was not just possible, but imperative.

### Positionality Statement

The authors of this article comprise a variety of neurotypes (we are a neurodiverse team) and have chosen to not disclose our individual disability statuses. The first and second authors are doctoral-level scholars and practitioners in the ABA field and have autistic and other-neurodivergent family members. The third and fourth authors are doctoral-level CAS scholars. One of the CAS authors completed a 40-hr registered behavior technician (RBT) training in order to gain better insight into ABA training practices. It should be noted that there is broad heterogeneity both inside the ABA field and inside the CAS field, so it is important to state explicitly that no members of the team attempted to represent their respective fields or neurotypes. The common goal uniting all four team members was to identify ways in which research and practice in ABA with autistic learners could change for the better through collaboration between ABA and CAS.

### Questions and Answers

During the initial meetings in which the team members laid the ground rules for collaboration, we agreed that each team create their own questions for the other side and email them to provide team members with ample time to compose calm, thoughtful responses to one another. After both sides received and read the answers to their questions, the team met again via video conference. The group engaged in unscripted discussion and came to a consensus that we worked well together and that we believed continued collaboration could be fruitful. We agreed to refine and clarify our answers where there were points of confusion. The responses by the CAS authors were subject to some small additions, shown in square brackets, after review to make them more accessible to ABA practitioners. More procedural details of this process are available in Suckle et al. (2025).

The nine questions posed to the CAS authors, and their respective answers, are organized under the following broader questions: (1) What are the criticisms of ABA from the CAS perspective? and (2) How can the fields of ABA and CAS work together to better understand each other with the objective of enhancing support for autistic people? The specific questions and answers are below.

#### What are the Criticisms of ABA from the CAS Perspective?

**Q1. ABA Authors’ Question:** What do you think about the criticism that ABA is fundamentally neurodiversity-denying, ergo if ABA was made neurodiversity-affirming, then it wouldn’t be ABA?

**A1. CAS Authors’ Answer:** This is wrong-headed criticism, as ABA fundamentals apply to behavior and not to either particular diagnoses or particular neurotypes. The circumstances where ABA can benefit a neurodivergent person are precisely those where it can benefit a neurotypical person. For instance, all children, whether autistic or not, can engage in tantrums but these should not be confused with autistic meltdowns due to cognitive and/or sensory overwhelm, which, unlike tantrums, are not problem behavior. Although mainstream ABA theory and practice surrounding autism currently is neurodiversity-denying because it fails to reflect differences between the neurotypes (e.g., autistic meltdowns, eye contact, and stimming), a neurodiversity-affirming ABA would be an ABA that acknowledges and accepts those differences. It would still be ABA but it would be autism centered in terms of its understanding of behavior.

Attempts to make ABA research and practice more broadly applicable to the needs of autistic people might take ABA further away from how ABA has traditionally been practiced. For example, full adoption of the neurodiversity paradigm necessitates greater focus on bidirectional social communication (nonautistic people have as much difficulty understanding autistic people as vice versa) and interaction difficulties, which should encourage more coaching around self-advocacy and critical positionality in relation to living and behaving as an authentic autistic person. This could be seen in terms of awareness and acceptance of behavior, validation of behavior, and recognition of social power relationships.

In summary, it is not just about asking how ABA can be neurodiversity-affirming but how ABA can evolve to the extent that it serves the needs of autistic people. It is also appropriate to ask whether, without the current infrastructure and monopoly of ABA in the U.S. context, there would be more freedom to evolve in ways that are both better for the autism support industry and better aligned with the needs of autistic people. In the end, whether it is called ABA or not, all support structures for autistic people need to serve, rather than traumatize, autistic people.

**Q2: ABA Authors’ Question:** One issue affecting the gulf between the ABA community and the neurodiversity community seems to be found in the fact that the underlying philosophy that forms the foundation for ABA (radical behaviorism, which is post-Watson), is largely unknown to the neurodiversity community and woefully inadequately known in the ABA practitioner community. For example, many ABA practitioners still believe that we can’t address emotions in ABA research and practice but this has been false since Skinner’s 1945 article “An Operational Analysis of Psychological Terms.” Does this sound relevant or would addressing this issue seem like a deflection from the issues that the neurodiversity community is mainly concerned with? In other words, ABA services can and should address autistic client’s emotions within the existing philosophical framework underneath ABA. But many practitioners don’t know this and so most remain neglectful on the topic of emotions. It is the job of the ABA community to better train our practitioners and many are working on it. So, would it be more productive for us to just keep working on this internally within the ABA field or could it be productive to dialogue on this with the neurodiversity community?

**A2: CAS Authors’ Answer:** What we appear to have is an ABA community (with honorable exceptions) that often not only fails to understand the behavioral differences between the neurotypes but surprisingly does not always fully understand the principles underlying ABA. We highlight the fact that much ABA training is focused solely on ABA-based techniques and fails to provide trainee ABA practitioners with any understanding of neurodivergence (this reflects the neurodiversity-denying status of mainstream ABA services for autistic people). But it appears that ABA training is also failing to provide trainees with all the necessary background to ABA practice. We agree that it is the job of the ABA community to train its practitioners. The ABA community should work with neurodivergent specialists to address the gap in relation to understanding of neurodivergence. This should be undertaken in parallel with addressing the gap in understanding of radical behaviorism. This would be an opportunity for ABA specialists and neurodivergent specialists to work together on both gaps. We think that a wider dialogue with the autistic community should cover all ABA gaps as it is clear that the possible achievement of a neurodiversity-affirming ABA is not solely dependent on acceptance of neurodivergent differences.

To speak to your example. Considering and addressing autistic clients’ emotions needs to be achieved through an autism-centered lens, with an appreciation that difficulties with interoception (picking up on internal bodily signals) and alexithymia (difficulties understanding and communicating one’s own and other’s emotions) might influence access to understanding and communicating emotions in particular in novel situations, with unfamiliar people, or when stressed. The needs of nonspeaking or minimally speaking participants in communicating emotions must be fully considered. Furthermore, it must be understood that for many autistic people the processing of emotions is delayed so interpretation and communication of emotions (verbally or nonverbally) may not be as immediate as neurotypical people expect.

If you are to produce genuine autism-supportive ABA research and practice, training on autism, involving autism specialists, is essential. Although we feel that this is an obvious point, we know that many ABA graduate programs and staff training programs do not currently involve autism training and that the BACB (2022) requires no specific training in autism. It seems a fundamental error in the legitimacy and regulation of the profession of ABA that ABA practitioners often work with autistic people despite having little or no understanding of autism. How can practice even start to be considered neurodiversity affirming, if no training in autism, the neurodiversity paradigm, and its implications has been included? At this point it is also apt to point out that we are aware of cases where ABA practice has capitalized on marketing services as “neurodiversity-affirming,” but where no substantial change, training in autism, or holistic realization of the neurodiversity paradigm takes place. These could be seen as instantiations of what Chapman (2025) refers to as “neurodiversity-lite,” and is a dangerous shift with intent to profiteer from, and abuse, a human rights movement.

**Q3: ABA Authors’ Question:** There is a huge quality problem in the practice of ABA. There is a huge gap between what the science and best practices look like and what actual practice in the community looks like. Is this gap relevant to the discussion? Or is this primarily an internal issue in the ABA field? On one hand it seems relevant because the vast majority of criticisms of ABA from the neurodiversity community seem to be about the practice of ABA in daily use, often in low-quality implementations. On the other hand, it seems not very relevant because if that’s the way ABA practice is done, then that is what matters.

**A3: CAS Authors’ Answer:** It is clearly important to differentiate between areas in which ABA approaches to practice are flawed and don’t serve the interests of autistic people and examples where ABA practice is *just* poor implementation of standard practice and not to conflate the two. It will be central to evolve standards and approaches that better support autistic people living fulfilling autistic lives. However, it will be important to ensure that consistent adherence with these standards is maintained across local practice and that deviations are subject to regulatory implications. It may also be commented that the quantity and extent of poor practice is likely the result of wider regulation problems including unchecked ethical practice, limited understanding of autism, and poor recognition and application of the values of the neurodiversity paradigm, which indicate need for greater oversight and further supervision through more centralized regulatory bodies.

**Q4: ABA Authors’ Question:** We address several criticisms of ABA in our 2024 article (ABA treatment programs seek to erase autistic identity, ABA services for autistic people harm mental health, ABA programs reduce whole human beings to behavior, autistic voices are absent in ABA research and practice, and ABA practitioners pressure parents to choose ABA). What are some additional concerns with ABA that we should have addressed and/or that future work in this area should address?

**A4: CAS Authors’ Answer:** The focus in your 2024 article (Mathur et al., 2024) was a very refreshing read and showed a frequently missing openness to engage in self-critical exploration on the history, shortcomings, and benefits of ABA services for autistic people. It indicated a commitment to engage with the neurodiversity paradigm to evolve practice.

For us there is a fundamental underpinning difficulty in relation to the evidence-base that ABA practice continuously refers to and uses to legitimize its methods and outlook. As much ABA service provision is practiced without explicit teaching on autism, or the neurodiversity paradigm, we query how it can be autism-centered? How can an effective functional analysis^1^ of behavior in autism take place if the practitioner doesn’t view that behavior through an autism lens? In addition, some so-called “problem behavior” is simply natural behavior of benefit to the autistic person. For instance, differences in eye contact in autism can enable an autistic person to focus better where it is difficult to integrate verbal and visual input. Likewise, a case can be made that the benefits and rationales underpinning stimming are often misunderstood by nonautistic people.

Greater focus should be placed on unravelling the short-term effects versus long-term impact with research into the outcomes for autistic people in the long term. As you rightly point out in your article, the long-term effects of misguided ABA services have been linked to post traumatic stress symptoms and there is crucial need to listen more carefully to the voices of autistic adults who have experienced ABA services as children in order to gain further insight into this. Once again, this is an area that is critically underfunded and unsupported and we note the criticism of Kupferstein’s 2018 unfunded study from voices within the ABA industry (Leaf et al., 2018; Dillenburger, 2025; Morris et al., 2025), without a concomitant commitment to support and fund further research into this vital area. We stress the need for further well-developed, transparent studies, co-produced with autistic scholars and the autistic community, into the link between ABA services and long-term mental health, including posttraumatic stress symptoms.

**Q5: ABA Authors’ Question:** The neurodiversity paradigm is an academic discipline that has been informed by many other disciplines, including Critical Autism Studies and disability studies. If we begin teaching ABA graduate courses, as well as train ABA practitioners, within a neurodiversity paradigm perspective, or consistent with this academic perspective, do you feel that would adequately address the criticisms of the neurodiversity community?

**A5: CAS Authors’ Answer:** We think that training from a neurodiversity paradigm perspective is necessary for the development of a neurodiversity-affirming approach to ABA research and practice but not sufficient. There is also a need for the neurodiversity paradigm to be adopted by ABA regulatory bodies, the ABA research community, and for treatment provision agencies to sign up to this paradigm. We would also wish to see the ABA community stop saying that ABA is the only evidence-based therapy for autism and being overtly critical of other interventions (often called eclectic interventions).

Neurodiversity-paradigm ABA training should be developed and delivered in conjunction with neurodiversity specialists, especially autism specialists. In addition, it is important to recognize that the rise of the neurodiversity paradigm contains two main messages: (1) that neurodiversity is an undisputable biological fact and (2) that neurodiversity is a minority rights movement. This emphasizes the need to shift from a medicalized deficit-based perspective on autism on the one hand but also to fully understand and respond to the socially constructed hierarchy in thinking around neuro-normative expectations on behavior and actions. As such, all teaching within ABA courses that address autism needs to start from an autism centered perspective and evaluation of support needs recognizing that such support needs stem from the barriers experienced in being autistic in a (most often) autism unfriendly environment. It is vital to not just teach neurodiversity in this sense but also to fully embrace its implications for action.

It should be acknowledged that not all autistic people support or favor the neurodiversity paradigm. The ABA industry needs to consider the different shareholders involved in each case but do so within the wider framework of recognizing that autistic people and parents of autistic children are likely to be influenced (and motivated) in their choices by the undisputed presence of societal barriers and inequities. These should always be the first point of redress. Much can be learnt in this regard from previous minority rights movements, such as those based on equal protection of rights irrespective of race, gender, or sexuality.

#### How can the fields of ABA and CAS work together to better understand each other with the objective of enhancing support for autistic people?

**Q6: ABA Authors’ Question:** There is a pretty significant problem with ABA researchers and practitioners not collaborating and dialoguing with people from other disciplines, not just CAS scholars (Slim & Reuter-Yuill, 2021). This gap exists between other equally problematic disciplines, such as psychiatry (Newhouse-Oisten, 2017). If ABA researchers and practitioners were respectfully dialoguing with professionals and scholars in other disciplines on a more regular basis, how might this help to move ABA forward to being more neurodiversity-affirming (cross-disciplinary collaboration in many fields to better support the needs of our clients)?

**A6: CAS Authors’ Answer:** Given that psychiatrists and psychologists are the professionals who diagnose autism and other aspects of neurodivergence it seems to us essential for the ABA community to enter into a dialogue with these professions, indeed with all professions who work with neurodivergent people in a therapeutic context such as speech and language therapists and psychotherapists. The psychiatric and psychology professions are beginning to take notice of the neurodiversity paradigm. We feel that this is a wonderful opportunity for the ABA community to join these professions (and others) on the same journey towards implementation of autism-affirming principles and practice.

It is also important to highlight that this is a broader problem where specialists in various areas of neurodivergence often fail to work together and in unison. Given the large co-occurrence of, for example, ADHD and autism, and ADHD and dyslexia, this is to the detriment of many individuals who would benefit from more joined-up diagnostic support, educational recommendations, autism affirming therapy, and medical care.

To respond to your question as to how such collaboration could move ABA research and practice forward to be more neurodiversity-affirming, one central way in which this would happen is that there would be better holistic understanding of the challenges and strengths an individual might experience in different contexts. Where focus narrowly adheres to a specific area, individuals who experience varying needs that necessitate an integrated response are ultimately failed. For autistic people who frequently present with uneven ability profiles there is also misunderstanding, neglect, and confoundment as to why support needs are high in some areas but not others. Collaborative dialogue and interaction between all shareholders could support better understanding of the dynamic nature of autism in different environments and at different time points in the individual’s life. This could help shape support that maps onto the bespoke, and potentially changing, needs across the lifespan.

It is our hope that this joint initiative between ABA scholars and CAS scholars will pave the way for greater collaboration and interaction between discordant voices on this topic. We feel that it is important to stress that even if consensus is not achieved, cross-disciplinary action and knowledge sharing is vital to further the perspectives of autistic people on all support structures (including ABA services). Autistic people need to be involved and listened to as co-producers of all support structures that concern them.

**Q7: ABA Authors’ Question:** What are some of the top behavior changes on the part of ABA folks that would make it clear to the autistic community that ABA folks want to affirm neurodiversity and are willing to change?

**A7: CAS Authors’ Answer:** We think that the following four “key” behavioral changes would demonstrate a genuine willingness to change on the part of the ABA community:

1. A wider and continuing dialogue between the ABA community and the autistic community demonstrating a commitment and genuine will on the part of the ABA community to change in ways that are meaningful to autistic people;
2. Adoption of the neurodiversity paradigm by the ABA community including regulatory bodies, providers, and researchers so that all interventions are viewed through an autism lens;
3. Functional analyses are autism centered i.e. demonstrate understanding of the reasons and purpose underpinning behavior and action through an autism lens;
4. Ethical matters are considered and reported on in all ABA research in autism including consent/assent and disclosure of conflicts of interest.

These changes necessitate the ABA community being accepting of the validity of autistic-led research and the opinions of autistic participants in that research. Only then could one say that autism research will achieve epistemological integrity.

**Q8: ABA Authors’ Question:** ABA folks are highly oriented to measuring change and outcome. How might we develop a measure for documenting the degree to which an autism support service produces a neurodiversity-affirming meaningful outcome? In other words, from a neurodiversity perspective, how do we know if a service was effective? It’s easy for us ABA folks to measure this in a nonstandardized, individual client way: ask the client what outcomes they want and then document whether we achieved those outcomes. But at broader levels, e.g., across a clinic, across multiple clinics, regionally, when looking at randomized clinical trials, there is no standard measure that exists, that we know of.

**A8: CAS Authors’ Answer:** This is an important question but not one that we can answer other than to say:

1. We are also unaware of any such measure; and
2. The development of a measure of ABA service outcomes for autistic individuals requires a (fully funded) project involving ABA practitioners, autism specialists, psychiatrists, psychologists, parents, carers, and—most important—autistic individuals who have undergone ABA treatment;
3. There is very little research on measurement of autistic well-being as opposed to well-being generally and that this gap must be attended to first.

It is also worth noting that it seems contradictory to us that ABA service can be practiced, with a view to improving autistic well-being, without meaningful conversations with autistic people and researchers on the topic of autistic well-being. In our searches, ABA academic research and publications have done little to redress the need for more focus on autistic well-being. Although your recent article (Mathur et al., 2024) goes some way to explore how particular ABA procedures might be detrimental to autistic well-being (focus on erasing autistic identity, compliance, and the reduction of autistic people to their overt behaviors), and several other articles query whether ABA treatment serves the well-being of autistic people (Sandoval-Norton et al., 2019), there is a desperate need for more research on what actually constitutes autistic well-being. Robust and well-funded research into autistic well-being needs to focus on how well-being is perceived by autistic people with an understanding that this will differ greatly across the heterogeneous autistic population.

We would add that ABA research and practice appears to focus on measuring what can be measured rather than what needs to be measured (i.e., improvement or deterioration in the lives and well-being of autistic people). For example, the use of the “quiet hands” technique may reduce the level of stimming that a child engages in and would be reported by ABA practitioners as a successful outcome. But if the child’s stimming is a calming mechanism or helps them to focus, ABA practitioners will have failed to measure the harm done to the child if the use of quiet hands leads to the child masking their natural behavior in future.

**Q9: ABA Authors’ Question:** Would it be beneficial to identify ways in which ABA research and practice addresses intersectionalities when working with autistic learners, so that we are considering the whole person and how different parts of their identity affect their experiences as a whole? Is there literature already out there in different fields on this topic?

**A9: CAS Authors’ Answer:** Although the issue of intersectionalities is important, there are some more fundamental issues to consider here. First, your question presupposes that there is a part of an autistic person’s identity which is not autistic when we regard autism as a different way of being. Second, we wonder why ABA services for autistic people often focus on behaviors that may be characteristic of an autistic person’s way of being, rather than on the aspects that would lead to a better quality of life. This suggests that ABA practitioners regard certain behaviors of autistic people as fundamentally defective. Does this explain why so much of the ABA service industry is focused on autism (and that some ABA providers are solely focused on autism?) Surely the behavior that would justify the use of ABA services would apply to all neurotypes? We believe it is essential to identify those aspects of behavior that may be appropriate for ABA interventions generally.

Furthermore, the focus of ABA services on behaviors that may be characteristic of an autistic way of being has to be understood within a socially constructed hierarchy of identities/behavior, which generally devalues behavior associated with one’s autistic identity (e.g., societal stigma against repetitive behaviors). This is highly problematic and at the root of much CAS critique of ABA. It is essential that ABA research and practice recognize the socially constructed environment around the autistic person and how this might influence how authentically autistic the person feels able to be in various settings. Race, culture, gender, sexuality will feature prominently in these decisions and should therefore be carefully considered in any support approaches. Again, research into racialized experiences of autism is woefully underfunded and although excellent work is taking place (see, for example, Brown et al., 2017) this is frequently not given enough focus and weight in these discussions.

## Discussion

The dialogue among the four authors of this article suggests that there may be scholars on both sides of the ABA/CAS divide who are willing to engage in productive and respectful conversations regarding how ABA research and practice can better serve the autistic community. Although this dialogue is centered on knowledge exchange rather than achieving consensus, it is promising to note that the CAS scholars believe there is opportunity for ABA to evolve in ways that could better serve autistic people (A1). It is important to note that many (although we do not have data to know how many) in the autistic community may be hesitant to trust ABA professionals and may need to see substantial evidence of BCBAs changing our practices in tangible ways in order to feel any degree of confidence that researchers and practitioners of ABA are ready to collaborate in good faith. It is also likely worth noting that members of the ABA community may find it hard to trust collaborating with scholars and activists in the autistic community who have overtly criticized the field of ABA in the past, perhaps even calling for ABA to be abolished. We recognize that peaceful and constructive dialogue will be uncomfortable, but we believe further dialogue will help ensure that autistic learners are respectfully supported and empowered to thrive. Furthermore, we believe that centering autistic voices in how ABA evolves will make the science and practice of ABA stronger because it will strengthen the moral and ethical foundation of our field (Allen et al., 2024).

The call from the neurodiversity movement for centering autistic voices when working with autistic people can be directly related to the concept of social validity, which is an important part of the moral and ethical foundation of ABA. Social validity was originally defined as the extent to which people *other than behavior analytic researchers* value the goals, procedures, and outcomes of behavioral intervention (Wolf, 1978). The “other people” in social validity have been variously referred to as “participants,” “consumers,” or “society” (Wolf, 1978). In the case of ABA supports for autistic learners, “consumers” and “participants” could be interpreted to mean either the autistic learners themselves, their parents/caregivers, their teachers/staff members, or other allied professionals, among others. Although ABA research has made some progress in assessing social validity from the standpoint of parents/caregivers (Ledford et al., 2016), ABA research and practice has seriously neglected assessing social validity from the standpoint of the autistic learners, who are of course the direct recipients of ABA services and supports (Hanley, 2010). Relying on what goals of intervention might be acceptable to parents and society at large may be inherently problematic if that society is biased or prejudiced against characteristics of the autistic clients one is intending to support (Chown & Murphy, 2022). Rather than relying heavily on what we believe parents or society would find acceptable for assessing social validity, we can move forward by centering autistic voices in how we assess goals, procedures, and outcomes of ABA supports intended for autistic learners. This shift has the potential to simultaneously strengthen our approach to social validity, while also addressing a key concern of the neurodiversity movement (Veneziano & Shea, 2023).

In order for the ABA field to evolve toward a more neurodiversity-affirming science and practice, ABA researchers and practitioners will need to take practical steps. The actionable steps presented in Table 1 were distilled from the CAS authors’ responses in this article.

**Table 1:** Actionable Steps in the Areas of General Collaboration, Training, and Research/Practice

**General Collaboration**
Engage in broader and more frequent dialogue between the autistic community and ABA community
Seek out autistic collaborators as equal co-creators of knowledge to orient research, practice, and measurement toward enhancing autistic well-being and quality of life
Increase collaboration with other professions (e.g., occupational therapy, speech therapy, psychologists, psychiatrists, autism specialists) to create a holistic understanding of support needs for each client
Don’t be overly critical of other interventions; ABA is not the only evidence-based therapy for autistic people anymore
Advocate for autism-specific ABA standards for research and practice developed in conjunction with the autistic community (and wider autism research and practice community).
Hire autistic experts on autism as consultants during program design and review efforts, at schools, clinics, and graduate programs
**Training**
Train ABA researchers and practitioners on the neurodiversity paradigm and specifically on autism
Receive training on emotional support needs through an autism lens, for example, challenges with interoception, alexithymia, and non-vocal communication of emotions
When planning supports, consider modifying the environment first, before assuming the autistic client’s behavior needs to be modified
Train ABA researchers and practitioners on radical behaviorism and/or functional contextual philosophy as a foundation for addressing autistic learners’ private events (i.e., emotions and cognitions)
Collaborate with autistic partners to revise and/or create new staff training programs on how to implement ABA supports for autistic learners
Incorporate multidisciplinary training on autism (not focusing primarily on diagnostic criteria) in staff training at every level (e.g., behavioral technician, case manager, clinical supervisor)
**Research/Practice**
Intervention targets should reflect what the autistic person wants, especially self-advocacy skills, to improve the quality of life for that person
Reevaluate whether behaviors traditionally labeled as “problem behaviors” in behavior reduction research may be part of autistic ways of being in the world
Collaborate with autistic scholars on research evaluating short- and long-term effects on quality of life, adverse events, and potential problematic outcomes of ABA services (e.g., PTSD symptoms)
Collaborate with autistic partners to revise and/or create new curricula for skills to include in ABA service programs for autistic learners

One of the central themes of the CAS authors’ responses is that ABA researchers and practitioners who are working with autistic learners must be trained on autism from an autistic lens. Indeed, early in the conversations that led to the dialogue in this article, they expressed dismay that, although 82.11% of BCBAs work with autistic people (BACB, n.d.), most ABA graduate programs and agency training programs do not teach about autism, beyond rudimentary diagnostic information. These concerns are reflected in a recent discussion article by Johnson (2025), in which the author called for behavior analysts to have a greater degree of training on autism during their graduate training experience, including differences in how minimally verbal autistic children might express distress. It is interesting to note that behavior analysis, as a science, has always emphasized the general applicability of behavioral principles of learning and motivation and has deemphasized the role of diagnoses, particularly how traditional medicine often treats diagnoses as causes of dysfunction (Follette et al., 1992). In general, this can be a strength of behavior analysis and is consistent with the social model of disability. However, it seems possible that, while trying to avoid fallacious aspects of focusing on diagnoses, the field of ABA may have inadvertently remained uninformed of the importance of understanding autism as a distinct neurotype. Focusing solely on skills and behaviors that seem important when viewed from the perspective of the predominant neurotype, without considering how those same skills and behaviors may be viewed by an autistic neurotype, may result in behavior analysts focusing on changing behaviors that don’t need to be changed, as well as not considering the importance of supporting other skills and behaviors that may be important from an autistic perspective. For example, some autistics may benefit from social skills such as refraining from small talk, telling the truth even when it is uncomfortable, and even sitting quietly in social situations, despite the possibility that some neurotypical people might not see these as social skills (Cage et al., 2024).

Although the *BACB Task List* (2022) cannot include items relevant to a particular neurotype because it is a general task list that outlines behavior analytic competencies, the ethics code does highlight the importance of practicing within our scope of competence. It is possible that behavior analysts have often thought of scope of competence as pertaining to practical experience in applying ABA principles and procedures with a specific population, not necessarily learning about that population. In response to concerns from the autistic community, it would seem reasonable for a behavior analyst’s scope of competence to include learning a large variety of multidisciplinary information about the population they serve, by collaborating with people from that population and especially by consuming information produced by authors from that population.

In addition to the need for service agencies to train practitioners on information about autism, graduate training programs have a large role to play. Of course, university programs with master’s programs in the general science and practice of ABA are not graduate programs on autism. Some graduate programs may lament that research and practice in ABA has become so focused on autism in recent decades and they may yearn to expand their students’ focus outside of autism, rather than emphasize a focus on autism. From the standpoint of providing comprehensive training in the science of ABA, a broad focus is laudable. However, given that 82.11% of the graduates from ABA master’s programs will be working with autistic clients (BACB, n.d.), it seems clear that graduate programs must play a larger role than they currently do in educating their students about autism. One strategy could be to build-in elective courses on autism from other university departments and programs, such as disability studies, speech pathology, occupational therapy, special education, among others. However, some program curricula may already be too full and requiring additional courses could also be cost prohibitive, especially for students coming from economically disadvantaged backgrounds.

An additional strategy for incorporating autism information into curricula of ABA master’s programs could be to reconsider how autism and examples of autism treatment are discussed throughout all courses in the existing curriculum. For example, when covering the responsibility to do no harm and to monitor for adverse effects of treatment in an ethics course, course instructors could have students read blogs or autobiographies from autistic authors who have had mixed experiences with ABA programs in the past. Or in an assessment course, when discussing how to prioritize selecting behavioral targets for intervention, course instructors might have students read source material by autistic authors who describe what it was like for them to have clinicians insist on eye contact during services. Likewise, instructors might lead discussions on choosing behavioral targets because they are characteristics of autism, versus choosing behavioral targets because they are likely to result in the autistic person accessing more of what they value in their daily lives. During a course on behavioral interventions, when discussing the social validity of interventions, course instructors might assign readings that address the social validity of various behavioral intervention procedures (e.g., physical guidance or escape extinction) written by autistic authors.

In addition to graduate programs taking greater responsibility for educating behavioral clinicians about autism, there may be a role for professional oversight organizations to play as well. In the past, organizations that seek to accredit service provision organizations have sometimes been for-profit companies with no transparency as to who owns them or how they are accountable to the profession more broadly. However, the Council of Autism Service Providers, a nonprofit professional association, founded the Autism Commission on Quality (ACQ). The ACQ is a nonprofit organization whose mission is to assess the quality of services provided by agencies according to a list of standards (ACQ, n.d.) and provide accreditation to organizations who meet those standards. Organizations such as the ACQ could consider requiring interdisciplinary information on autism, above and beyond autism characteristics (symptoms in the *Diagnostic and Statistical Manual *(American Psychiatric Association, 2013)), in organizational staff training.

### Implications for Future Research

Several of the main points made in the CAS authors’ answers have clear implications for future research on ABA services for autistic people. A general theme that seems to cut across many points is that ABA researchers could benefit from being more open to a variety of viewpoints throughout the research process, starting from identifying research questions that matter, all the way up to evaluating the long-term effects of services. In this context, it might be worthwhile for the ABA field to regularly reconsider why we choose specific behaviors to conduct research on. To what extent has our foci in autism service research been influenced by our own traditions and to what extent have they been influenced by calls from other critical stakeholders, including autistic adults who have received ABA services in the past, as well as professionals from other disciplines who support autistic people? To what extent has expert information on autism from outside ABA influenced how we view and select target behaviors for assessment and reduction?

One possible direction for reevaluating autism-focused ABA research would be for ABA researchers to invite autistic people and other experts in autism as genuine collaborators in the research process, from conceiving research ideas to co-authoring articles. A brief commentary by Jackson-Perry et al. (2025) suggest such an approach, referring to the creation of an interdisciplinary, collaborative field of study deemed *Critical Behavioral Studies.* Space does not permit a thorough discussion of the details of creating a new field of inquiry in this article, but it is worth noting that such collaboration seems possible. Future researchers will need to try such collaborations and see what type of research they spawn. At a minimum, such collaborations might start with ABA researchers identifying colleagues from other disciplines, such as Critical Autism Studies, disability studies, and others, as well as autistic advocates, to form a team of equal co-investigators. The minimum qualifications of all team members would be that they believe it is possible for ABA to do better in the future and they are willing to work together, in a mutually respectful and collaborative manner toward that goal. Such teams might identify specific topics and systematically evaluate historical and sociopolitical factors, previously published evidence, and discuss current concerns. They could then identify specific research questions that have not been researched sufficiently and design, execute, and publish studies to address the research questions.

In addition to taking practical action on the steps outlined above, we have much to consider in the field of ABA supports for autistic people. Many more opportunities for change likely exist and many issues likely do not have clear solutions. Put simply, the CAS authors’ answers challenge our field in ways that we may not know how to practically address at this moment. Therefore, we propose potential points for continued reflection in Table 2.

**Table 2:** Points for Reflection

Consider that traditional ABA concepts (e.g., four common functions of behavior) may not capture some variables that are critically important to the autistic experience. Are there other concerns that autistic advocates voice, which are not adequately encapsulated by traditional ABA concepts and are we willing to listen with curiosity?
Consider which problems within our current service landscape are created by low-quality implementation and which practices may be problematic because they are not autism-affirming, regardless of quality
Before addressing a behavior with ABA procedures for an autistic client, ask if that behavior would be addressed with all neurotypes. For example, would we work on that same behavior with a neurotypical child, and if not, why are we working on it with a neurodivergent child?
Consider the implications of the social model of disability for our practice, including that there exists a hierarchy of identities and behaviors that are socially constructed. Autism is a minority neurotype, and like other minority rights movements that were based on equal protections of rights (e.g. race, gender, sexuality) we must note the cultural, social, and political environment of an autistic person and how these social variables assign values to behavior. This can mean that behavior has a different meaning depending on the neurotype of the person engaging in it or the person assessing it.

No attempt at dialogue is without limitations. Perhaps the single largest limitation of the dialogue described in the current article is that it did not include the opinions of nonspeaking or minimally speaking autistic people. Because a substantial minority of autistic people are nonspeaking or minimally speaking, it seems important to have their needs and perspectives represented in dialogues on autism. Future collaborations might consider including family members of nonspeaking autistic people, others with substantial lived experience working with and/or caring for them, as well as including accommodations in dialogues to better support the communication needs of nonspeaking autistic collaborators.

## Conclusion

Meaningful collaboration between the fields of ABA and CAS with the aim of improving ABA services for autistic clients will require continued commitment on the part of both disciplines. At best, this article may serve as an initial step to spur others to move forward collaboratively. Indeed, we hope that future researchers and practitioners will consider the practical steps and points of reflection posed above and improve upon, expand, and elaborate on further collaboration. This article, along with its partner article, Suckle et al. (2025), exemplifies how two different fields can successfully engage in the exchange of knowledge to help both fields understand different perspectives. This may be a rare occurrence in academia, where interdisciplinary collaboration is infrequent and respectful dialogue about criticisms of one's field are difficult. We hope that further collaboration, with a broader team of collaborators, will continue to help evolve ABA research and practices toward being more autism-affirming, compassionate, and consistent with the behavior analytic core values of being noncoercive and socially valid.

## Footnotes

1 We note that the standard behavior types in an ABA functional analysis are “access to tangible items,” “escape/avoidance,” “attention-seeking,” and “automatic reinforcement.” Without further elaboration from the autistic person’s perspective, the category of “automatic reinforcement” does not apply to eye contact or stimming in autism (these are just examples). Current ABA functional analysis is inadequate for understanding automatically reinforced behaviors from the autistic perspective.
